# Intertemporal Improvement in Physicians’ Perceptions of the Short-Term Adverse Outcomes of Neonatal Pain: Results of a Two-Time-Point National Survey

**DOI:** 10.3390/children11040471

**Published:** 2024-04-15

**Authors:** Eleni Agakidou, Angeliki Kontou, Theodora Stathopoulou, Maria Farini, Agathi Thomaidou, Konstantina Tsoni, William Chotas, Kosmas Sarafidis

**Affiliations:** 1 Department of Neonatology and Neonatal Intensive Care, Faculty of Medicine, School of Health Sciences, Aristotle University of Thessaloniki, Hippokrateion General Hospital, 54642 Thessaloniki, Greece; angiekon2001@yahoo.gr (A.K.); farinimaria@yahoo.com (M.F.); kosaraf@auth.gr (K.S.); 2 Department of Neonatology, University of Vermont, Burlington, VT 05405, USA

**Keywords:** adverse effects, analgesics, cardiovascular, central nervous system, neonatology, preterm infants, outcome, pain consequences, respiratory system, sedatives

## Abstract

Pain in early life may seriously impact neonatal outcomes. This study aimed to evaluate whether the perceptions of physicians working in neonatal intensive care units (NICUs) of the short-term adverse outcomes associated with neonatal pain have changed over a 20-year period. Self-administered questionnaires were distributed to 117 and 145 neonatologists, pediatricians, and fellows working in level III NICUs in 2000 (T1) and 2019 (T2), respectively. The questionnaire consisted of four domains, including the central nervous, cardiovascular, and respiratory systems, as well as “other systems” (metabolic/endocrine system, growth, and general condition), with 21 total items overall. Although the proportion of positive (correct) responses to the total and system-specific domain scores was significantly higher at T2 than T1, the knowledge of certain short-term adverse outcomes was suboptimal even at T2. Adjustment for cofactors confirmed the independent association of the survey time-point with the total and system-specific domain scores. Moreover, NICU type was an independent significant factor associated with the adjusted total and central nervous system scores, while young doctors had a better knowledge of adverse cardiovascular effects. Conclusions: The perceptions of NICU physicians concerning the short-term outcomes associated with neonatal pain have significantly improved over the past 20 years, although remaining knowledge gaps mandate ongoing efforts to achieve an improvement in neonatal care.

## 1. Introduction

Neonates, especially those born prematurely, have been well documented not only to perceive pain but also to have a lower pain threshold and tolerance compared to older children and adults [1]. Moreover, a growing body of evidence has documented that pain has acute adverse physical, physiological, and behavioral effects on developing neonatal organs—or systems [2,3]. Physiological responses to pain are generally categorized according to the organ system affected, such as the nervous system or cardiorespiratory system, or according to the metabolic or hormonal alterations observed [4,5]. In addition, repeated exposure to painful stimuli during critical developmental stages of life, such as the intrauterine and early postnatal periods, may cause serious structural and functional changes in the central nervous system (CNS) [1,6].

Educational programs and the development of clinical protocols can improve the knowledge on neonatal pain among healthcare professionals, leading to more effective management. In this context, during the last four decades, relevant lectures and courses have consistently been included in scientific programs of neonatal/perinatal congresses at both the national and international levels. Additionally, a large number of published scientific articles specifically focus on the prevention, assessment, and treatment of neonatal pain and discomfort, and recommendations have been issued by national and international scientific societies [7,8]. 

Relevant studies have shown that the great majority of health professionals do believe that neonates are capable of feeling pain and that pain must be effectively treated [9,10]. However, it was also found that the existing scientific evidence is not consistently applied in clinical practice, and the use of analgesia is not always employed during painful procedures [11,12,13,14]. Consequently, the assessment, prevention, and treatment of neonatal pain remain suboptimal [12,15,16]. Difficulties in recognizing pain in neonates, a lack of experience in the use of pain assessment tools, and a shortage of staff, as well as the limited availability of unit protocols and/or firm guidelines, are some of the factors contributing to inefficient management of neonatal pain [12,15,16]. 

Understanding the adverse effects pain may have on neonatal outcomes is a crucial step towards its prevention and management. We hypothesized that the unsatisfactory level of knowledge and awareness of the adverse effects of pain among professionals working in neonatal intensive care units (NICUs) may be an additional factor contributing to its suboptimal management in neonates. To the best of our knowledge, despite the accumulated evidence over the past 40 years, available data regarding the intertemporal changes in the perceptions of NICU physicians of the short-term outcomes of neonatal procedural pain and stress are missing. The aim of this study was to evaluate whether neonatologists’ perceptions, concerning the short-term outcomes that have been associated with neonatal pain, have changed over the past 20 years.

## 2. Material and Methods

### 2.1. Study Design

This prospective study was part of a national survey conducted at two time-points, in the years 2000 and 2019, aiming to investigate the perceptions and clinical practices regarding neonatal pain and stress of Greek physicians caring for high-risk neonates [10]. The current part of the survey focused on the physicians’ perceptions of the short-term adverse outcomes potentially associated with neonatal pain. 

### 2.2. Study Population

The candidates for inclusion in this study were all physicians working in level III neonatal intensive care units (NICUs) all over Greece in the years 2000 (time-point 1, T1) and 2019 (T2). This included certified neonatologists, certified pediatricians who had worked in NICUs for at least three years, and pediatricians that had attended a fellowship program in neonatology for at least five months. The study population was divided into two time-point-related main groups; Group T1 consisted of the respondents in 2000, and Group T2 consisted of the respondents in 2019. 

### 2.3. Methodology

Self-administered questionnaires were distributed to the study population via the directors of 13 and 15 NICUs’ at T1 and T2, respectively, as previously described [10]. Besides the demographic characteristics of the participants, the questionnaire consisted of four domains, including adverse effects that have been previously reported in four organ/systems: the CNS (4 items), cardiovascular system (CVS, 6 items), respiratory system (8 items), and an “other” systems category, which included 3 items (metabolic and endocrine effects, growth, and deterioration of infants’ general condition) (the questionnaire is shown in Supplementary Table S1). The answers to each single question (item) were in categorical format (YES/NO). Missing answers were not included in the analysis. The questionnaire was constructed based on published data regarding the potential short-term effects of pain in early life and was reviewed by experienced neonatologists (the current directors of the NICUs included in the study) for potentially ambiguous questions. The reviewers’ suggestions were considered for the construction of the final version of the questionnaire. Each correct (YES) answer to the items was given a score of 1, while a negative (NO) response was given a score of 0. The domain scores consisted of the sum of the respective item scores. The sum of the four system-specific domain scores was also calculated (herein referred to as the total score). The highest possible total score for each participant was 21. 

All the work was conducted in accordance with the Declaration of Helsinki. The study did not need approval from the Review Board of our institution, as the questionnaire used did not refer to patients but to doctors from almost all the NICUs in Greece who freely expressed their perceptions without using any hospital data or records. Moreover, the questionnaire was anonymous, and the respondents could not be identified from the limited demographic data included in the questionnaire. An explanatory invitation letter and consent form was attached to the questionnaire. The participants were instructed to sign the consent letter and return it with the completed questionnaires. 

### 2.4. Data Analysis

Dichotomous data were expressed as counts and proportions and continuous data as medians and interquartile range, as they were not normally distributed (Kolmogorov–Smirnov test). Comparisons were performed using Fisher’s exact test and the Mann–Whitney U test, as appropriate. The sum scores for each domain and the total sum score, as well as the percentage changes in the total score between T1 and T2, were calculated. The associations between continuous variables (i.e., years of work in an NICU vs. total score and system-specific domain scores) were assessed using Spearman’s coefficient of variation. Multiple regression analysis models were constructed to investigate the association of the outcome measures (i.e., total domain score, and system-specific domain scores) with the time-point of the survey after controlling for potential cofactors, including the NICU type (i.e., public vs. private hospitals), as well as the participants’ sex, expertise level (certified neonatologists/pediatricians working in NICUs for at least 5 years vs. fellows), and years of work in NICU. The analyses were performed using general linear models ( multivariate model). The limit of significance was set at *p* = 0.05. The IBM SPSS software (version 23) was used for the data analysis.

## 3. Results

### 3.1. Demographic Data

Questionnaires were distributed to 117 and 145 physicians at T1 and T2, respectively. The response rates to the part of the survey regarding the short-term adverse effects of pain at T1 and T2 were 88.9 (*n* = 104) and 77.2% (*n* = 112), respectively. During the 20 years that passed between the two time-points of the survey, a considerable proportion of the NICU physician population changed, mainly due to the retirement of several T1 participants. Nevertheless, the two time-point-related groups were comparable regarding their sex distribution, the proportions working in public vs. private NICUs, their expertise level, and their time spent in the NICUs (Table 1). At both time-points, most of the participants (75%) were either certified neonatologists or experienced pediatricians working in level III NICUs (Table 1). Moreover, the duration of work in the NICU was significantly longer for the neonatologists/pediatricians than for the fellows, as was expected (*p* < 0.001, Table 2).

### 3.2. Physicians’ Perceptions

Table 3 shows the numbers and proportions of respondents who gave positive responses to the items of the four domains. A significantly higher proportion of respondents in Group T2 responded positively to almost all individual items than Group T1 (*p* < 0.001 for all comparisons, Table 3). Only tachycardia and heart rate fluctuations were considered pain-associated comparably between the two groups (Table 3). 

**Figure 1:**
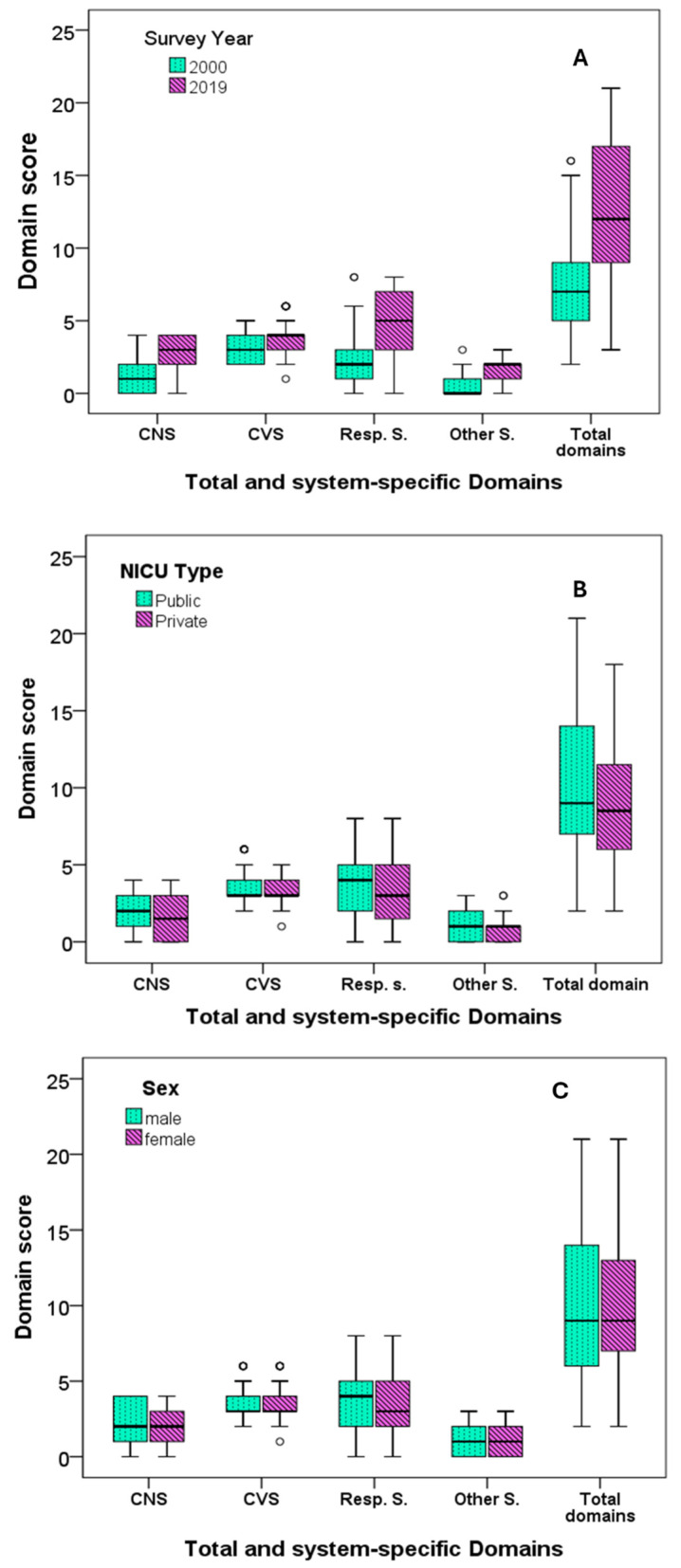

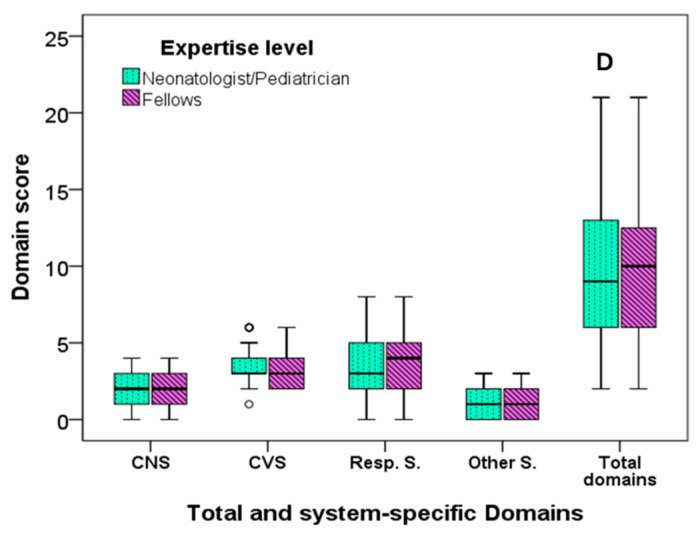
Total and system-specific domain scores in relation to the time-points of the survey: (**A**), NICU type (**B**), sex (**C**), and expertise level (**D**). All differences in the scores between the T1 and T2 groups were significant at *p* < 0.001. There was no other significant difference; CNS, central nervous system; CVS, cardiovascular system; s, system; resp, respiratory system.

Comparisons of the scores between T1 and T2 showed that the total and system-specific domain scores increased significantly from T1 to T2 (*p* < 0.001 for all comparisons) (Table 2, Figure 1A). Regarding the participants with total scores equal to or less than the fifth centile of all the respondents from both T1 and T2, there were 30 (29%) participants at T1 and 8 (7%) participants at T2 (*p*< 0.001). The highest score for Group T1 was 16 (attained by two respondents), and the highest score for Group T2 was 21 (attained by six respondents). A total score higher than 9, which was the 50th centile of the scores of all the respondents from both T1 and T2, was obtained by 16% and 71% of the T1 and T2 participants, respectively (*p* < 0.001). The scores were further compared between subgroups related to NICU type, sex, and expertise level. No differences in the total and system-specific domain scores were found between the subgroups related to the NICU type and responders’ expertise level (Table 2, Figure 1B–D).

Spearman’s correlation coefficient did not show any significant correlation between the years spent working in an NICU and either the total or system-specific scores. 

The percentage change in the physicians’ perceptions of pain over the 20 years of the study is shown in Table 4.

### 3.3. Multiple Regression Analysis Results

We assessed the independent association of the total and domain scores with the year of the survey after adjustment for potential cofactors (NICU type and respondents’ sex, expertise level, and working years in NICU). The year of the survey was found to be a significant independent predictor of the adjusted total score and all the system-specific scores (Table 5). In addition, NICU type was significantly associated with the adjusted total domain score and the scores for the CNS domain (Table 5). The respondents’ sex was significantly associated with the CNS domain score after controlling for cofactors. The respondents’ expertise level showed a significant association with CVS score and a borderline association with the total domain score (*p* = 0.066). In contrast, working years in NICU were not associated independently with either the total score or any system-specific scores.

## 4. Discussion

The perceptions of health professionals caring for sick neonates surrounding the potential short-term consequences of pain have a great impact on the quality of care provided in an NICU setting [9,10,14]. In the current study, conducted at two different time-points 20 years apart, physicians working in NICUs were asked to specifically report their views concerning the short-term adverse outcomes potentially associated with neonatal pain, with the goal of evaluating the extent of the physicians’ knowledge on this topic and identifying whether changes had occurred over the time period. Indeed, we found significant differences between the two time-points in the survey, with an overall intertemporal improvement. A significantly higher proportion of the 2019 respondents correctly reported specific short-term adverse outcomes associated with neonatal pain. Moreover, the physicians at T2 had more in-depth knowledge, as the domain and total scores were significantly higher in the 2019 group before and after adjustment for cofactors. Interestingly, of the demographic factors, only the NICU type, but not the respondents’ sex, expertise level, or duration of work in an NICU, was identified as a significant independent factor associated with the adjusted total score and the scores for certain domains.

Pain has been associated with adverse effects and poor outcomes in different organ systems, with the CNS, CVS, and respiratory system being the most vulnerable. This is not surprising considering the decreased pain threshold of preterm infants and also the prolonged and enhanced painful stimuli they are exposed to during their NICU stays [4,9,17,18]. The reported short-term side effects on the CNS include large fluctuations in cerebral blood flow that augment the risk of cerebral hemorrhage, increased intracranial pressure, and stress—with associated hormonal derangements [6,12,17,19,20]. At the first time-point of our study (year 2000), only about half of the respondents were aware of the detrimental effects of pain on the CNS. This proportion and the score for the CNS domain increased significantly by 2019. Of note, only 25% of the T1 respondents were aware of the adverse effects of pain on cerebral blood flow, while this proportion increased by 268% (up to 92%) at T2. This increase in the neonatologists’ awareness could be attributed to the scientific knowledge obtained over the past two decades from using Near-Infrared Spectroscopy to monitor brain oxygenation and perfusion (cerebral blood flow) in neonates undergoing painful procedures [21]. Interestingly, the highest frequency of positive responses at both time-points referred to the amplifying effect of pain on stress responses.

The CVS is another system of interest, which is frequently affected by neonatal pain. The most common effects of painful procedures on the CVS include arterial hypertension, tachycardia, bradycardia, and increased heart rate variability [5,22,23,24]. In addition, changes in heart rate and arterial pressure following procedural pain can lead to fluctuations in cerebral blood flow and oxygen delivery [25]. Our study showed that the level of knowledge of the NICU physicians about the spectrum of certain cardiovascular effects of pain was extremely low in 2000. It is encouraging, though, that a significant increase in the positive response rate was observed in 2019 regarding all the components of the CVS domain. In 2019, arterial hypertension, tachycardia, and heart rate fluctuations were the most stated short-term adverse effects of pain, while knowledge on the risk of circulatory collapse remained extremely poor. The proportion of the respondents who considered bradycardia a pain-related sign was moderate at T1 and did not change significantly over the 20 years of the study period. The low awareness of pain-related bradycardia is an alarming finding given that bradycardia has been associated with marked changes in cerebral blood flow and oxygenation, and it can lead to life-threatening complications [25].

Various adverse effects of pain on the respiratory system have been described [3,4,12,24,26]. In our study, desaturations, ventilator asynchrony, hypoxemia, and apneic spells were the most frequently stated acute complications of neonatal pain at both time-points. It is worth noting though that despite the known association between hypoxemia and pulmonary hypertension [27], the latter was reported as a short-term complication of neonatal pain only by a small proportion of the respondents. The frequency of positive responses on the effects pain may have on breathing patterns, respiratory distress, pneumothorax, or the duration of mechanical ventilation was also low at T1 and did not reach a satisfactory level by 2019, despite a significant increase over time.

Overall, knowledge of the cardio-respiratory responses to pain is crucial for the identification of neonates in pain and to improve the quality of their management. Therefore, heart rate fluctuations, arterial pressure, oxygen saturation, and respiratory pattern are components of almost all the multi-dimensional tools proposed for the assessment of neonatal pain [12].

The domain “other systems” included metabolic and endocrine complications, as well as potential effects on growth and the infants’ general condition. Early studies documented the effect of pain on stress hormones (mainly catecholamines and cortisol) not only during painful procedures but also later in childhood and adult life [28,29,30,31]. The long-term effects of pain and stress on the endocrine and metabolic systems have been attributed to reprogramming of the hormone system and stimulation of the hypothalamic–pituitary–adrenal axis [28,29,30,31,32,33]. Regarding the effects pain has on early growth, studies in preterm infants have shown that the frequency of skin-breaking procedures in early life was negatively associated with postnatal weight and head circumference after controlling for confounding factors [28]. This effect could be the result of pain-induced energy expenditure and increased needs in terms of energy intake. This is particularly important for preterm infants, who have low energy reserves and increased catabolism. In addition, hormonal responses to pain secondary to the stimulation of the hypothalamic–pituitary–adrenal axis, such as cortisol and growth hormones, may adversely affect growth as well [28,30,31]. A low growth rate in early postnatal life, which is usually followed by quick catch-up growth, along with the stimulation of the hypothalamic–pituitary–adrenal axis, predisposes neonates to non-communicable adult diseases, such as hypertension and metabolic syndrome, which are more common in individuals born prematurely [12,24,28,34]. The results of the current study showed that the knowledge surrounding the endocrine, metabolic, and growth consequences of neonatal pain was extremely poor at T1 and remained poor at T2, despite the significant increase observed between the two time-points of the survey. 

It has been reported that doctors who have worked in neonatal units for a longer period of time are more likely to have a good knowledge of the consequences of pain [14]. In our study, the bivariate comparisons did not show any significant difference in the total and system-specific domain scores related to expertise level or years of working in an NICU. However, adjustment for cofactors revealed expertise level as a significant independent factor associated with knowledge of adverse effects on the CVS, which, interestingly, was greater in the fellows than in the more experienced respondents. In addition, adjustment for cofactors showed that the type of NICU—public vs. private—was a significant factor independently associated with the adjusted total score and certain domain scores, with the physicians working in public NICUs achieving higher total scores. Overall, these findings are reassuring and suggest that junior doctors working in NICUs become aware of the short-term consequences of pain soon after they start working in an NICU. Practical teaching by more experienced staff in public hospitals may have contributed to this difference. These findings are consistent with the overall increased awareness of pain that is discussed in neonatal academic circles and that has found its way into pediatric residencies and even medical school education.

The main factors that have probably contributed to the significant increase in knowledge observed in 2019 include (a) continuing medical education possibly due to easier access to international publications (especially to open access journals) and frequent pain-related lectures at international and national congresses, as well as intradepartmental educational programs and discussions during ward rounds [10]; (b) the accumulated evidence during the last 20 years suggesting that the risks of untreated pain outweigh those of medications [14]; (c) the extended use of monitoring in clinical practice that has allowed the direct observation of the acute physiological responses to pain; and (d) the increased use of pain assessment tools [10]. Whether the current study’s demonstrated improvement in the NICU physicians views’ is reflective of an improvement in clinical practice is an important issue. In our previous publication about the changes in the perceptions and practices of physicians working in NICUs over 20 years (from 2000 to 2019), we found a significant improvement in the physicians’ perceptions regarding the capability of neonates to feel pain and the treatment practices surrounding neonatal pain/stress, which include its prevention and early, prompt treatment [10]. Presumably, the changes in perceptions concerning the potential adverse effects of neonatal pain and stress found in the current study contributed to the positive impact on the treatment practices reported previously [10]. This is the only existing survey in which the intertemporal changes in physicians’ perceptions of the short-term adverse outcomes associated with neonatal pain were explored in detail. The obtained data could serve as an impetus for further improvement in medical education and ultimately neonatal care. The study’s limitations include the small number of participants from a single European country, and therefore our results cannot be generalized. Moreover, it was a questionnaire-based survey, which may show inherent “response bias”, adversely affecting the accuracy and reliability of the results. However, the fact that the participants could not be identified and that the results overall were far from “favorable” is rather revealing of the true level of knowledge and closer to reality.

In conclusion, the results of the current study indicate a significant intertemporal improvement in physicians’ knowledge regarding the short-term adverse outcomes associated with neonatal pain. However, there are still knowledge gaps which necessitate ongoing efforts to improve the early prediction of pain development and intensity, which, in turn, will help lead to pain prevention and the optimization of its management. The use of modern diagnostic tools, easily applied and requiring less time expenditure in clinical practice, may mitigate the short-term consequences of neonatal pain. In this context, artificial intelligence systems gathering multiple variables from NICU patients, including history, clinical, behavioral, and physiological changes and other parameters, are currently under investigation [35,36]. Nevertheless, no artificial system can substitute for the education, experience, and sensitivity of health professionals. 

## Figures and Tables

**Table 1:** Demographic data of the respondents.

	Total	2000	2019	*p* *
No	217	104	113	
**Sex**				0.373
Male (*n*, %)	64 (29.6)	34 (32.7)	30 (26.8)	
Female (*n*, %)	152 (70.4)	70 (67.3)	82 (73.2)	
**Type of NICU**				0.846
Public (*n*, %)	186 (86.1)	89 (85.6)	97 (86.6)	
Private (*n*, %)	30 (13.9)	15 (14.4)	15 (13.4)	
**Expertise level**				0.942
Neonatologists (*n*, %)	162 (75.0)	77 (74.0)	85 (75.9)	
Pediatricians (*n*, %)	15 (6.9)	8 (7.7)	7 (6.3)	
Trainees (*n*, %)	39 (18.1)	19 (18.3)	20 (17.9)	
Working years in NICU **	10.5 (14.5)	12 (14.0)	9 (15.0)	0.899 ^#^

* Fisher’s exact test; ** median (interquartile range); ^#^ Mann–Whitney U test.

**Table 2:** Years of work in NICUs, total score, and system-specific scores (median, interquartile range) in relation to the year of survey, sex, type of NICU, and expertise level.

	Year of Survey			Type of NICU			Sex			Expertise Level		
Domain Scores	2000	2019	*p* *	Public	Private	*p* *	Male	Female	*p* *	Neonatologists/ Pediatricians	Trainees	*p* *
Years of work in NICU	12 (14)	9 (15)	0.377	10 (13)	14 (15)	0.164	7 (12)	12 (14)	0.059	14 (13)	1.5 (1)	<0.001
Total score	7 (4)	12 (8)	<0.001	9 (7)	8 (6)	0.149	9 (8)	9 (7)	0.887	9 (7)	10 (7)	0.671
**Domain scores**												
Central nervous system	1 (2)	3 (2)	<0.001	2 (2)	1.5 (3)	0.097	2 (3)	2 (2)	0.507	2 (2)	2 (2)	0.728
Cardiovascular system	3 (2)	3 (2)	<0.001	3 (0)	3 (1)	0.334	3 (1)	3 (1)	0.891	3 (1)	3 (2)	0.183
Respiratory system	2 (2)	5 (4)	<0.001	4 (3)	3 (4)	0.260	4 (3)	3 (3)	0.963	3 (3)	4 (3)	0.377
Other systems	0 (1)	2 (1)	<0.001	1 (2)	1 (1)	0.494	1 (2)	1 (2)	0.939	1 (2)	1 (2)	0.608

* Mann–Whitney U test.

**Table 3:** Number (percentage) of positive responses to the question “Do you agree that neonatal pain may be associated with the following short-term adverse outcomes?”

No of Domain/Item	Domains and Items	Group T1	Group T2	*p* ^a^	OR	95% CI	
		(*n* = 104)	(*n* = 113)			Lower	Upper
**1**	**Total CNS**	61 (55)	104 (90)	<0.001	0.157	0.075	0.329
1.1	Change in cerebral blood flow	25 (24)	92 (80)	<0.001	12.320	6.487	23.414
1.2	Cerebral hemorrhage	14 (14)	53 (46)	<0.001	5.373	2.749	10.529
1.3	Increased intracranial pressure	35 (34)	64 (56)	0.002	2.402	1.386	4.163
1.4	Increased stress responses	57 (56)	96 (83)	<0.001	3.989	2.128	7.478
**2**	**Total cardiovascular**	80 (78)	109 (94)	0.001	0.200	0.078	0.516
2.1	Hypertension	30 (29)	97 (84)	<0.001	12.933	6.691	25.001
2.2	Tachycardia	76 (74)	98 (85)	0.060	1.972	0.998	3.896
2.3	Bradycardia	30 (29)	59 (51)	0.001	2.529	1.442	4.433
2.4	Heart rate fluctuation	51 (50)	105 (91)	<0.001	0.095	0.045	0.203
2.5	Pulmonary hypertension	13 (13)	32 (28)	0.007	2.639	1.297	5.372
2.6	Circulatory collapse	7 (7)	19 (16)	0.036	2.686	1.079	6.685
**3**	**Total respiratory**	76 (73.8)	113 (100)	<0.001	0.050	0.012	0.216
3.1	Irregular breathing pattern	11 (11)	73 (63)	<0.001	14.537	6.995	30.210
3.2	Apneic spells	36 (35)	83 (72)	<0.001	4.827	2.717	8.577
3.3	Respiratory distress	7 (7)	43 (37)	<0.001	8.190	3.482	19.264
3.4	Oxygen desaturations	61 (59)	96 (83)	<0.001	3.270	1.756	6.092
3.5	Hypoxemia	39 (38)	86 (75)	<0.001	4.896	2.726	8.686
3.6	Ventilator asynchrony	54 (52)	85 (74)	0.001	2.570	2.571	4.538
3.7	Increased duration of mechanical ventilation	10 (10)	44 (38)	<0.001	5.763	2.715	12.237
3.8	Pneumothorax	17 (16)	47 (41)	<0.001	3.497	1.845	6.627
**4**	**Total other systems**	31 (30)	84 (73)	<0.001	0.159	0.088	0.286
4.1	Deterioration of the clinical condition	20 (19)	76 (66)	<0.001	8.087	4.340	15.070
4.2	Hormonal and metabolic derangement	18 (17)	65 (56)	<0.001	6.139	3.276	11.505
4.3	Slow growth rate	6 (6)	47 (41)	<0.001	11.174	4.523	27.607

^a^ Fisher’s exact test; CNS, central nervous system.

**Table 4:** Percentage changes (median, interquartile range) of the total and specific-system domain scores from T1 to T2.

	% Changes
Total score	100 (151)
CNS score	33 (100)
Cardiovascular score	33 (50)
Respiratory score	67 (166)
Other system score	25 (125)

**Table 5:** Multiple regression analysis of the factors significantly independently associated with the adjusted scores of the four system-specific domains and the total score (general linear model—multivariate model).

Dependent Variables (Domain Scores)	Significant Independent Variables	B	Sig.	95% Confidence Interval		Observed Power
				Lower Bound	Upper Bound	
CNS	Survey year	−1.518	<0.001	−1.885	−1.151	1.000
	NICU type	0.553	0.036	0.035	1.070	0.554
	Sex	0.423	0.045	0.009	0.837	0.519
CVS	Survey year	−0.760	<0.001	2.583	3.671	1.000
	Expertise level	0.578	0.015	0.114	1.043	0.685
Respiratory system	Survey year	−2.802	<0.001	3.228	5.246	1.000
Other systems *	Survey year	−1.258	<0.001	0.783	1.719	0.999
Total domain sum score	Survey year	−6.337	<0.001	8.560	12.569	1.000
	NICU type	1.748	0.037	0.110	3.386	0.554
	Expertise level	1.607	0.066	−0.106	3.321	0.453

Independent variables included in the model: survey years; NICU type (public vs. private); and respondents’ sex, expertise level (certified neonatologists/pediatricians vs. fellows), and years of work in NICU. * “Other systems” includes three items: metabolic and endocrine effects, growth, and deterioration of infants’ general condition; CNS, central nervous system; CVS, cardiovascular system.

## Data Availability

The original contributions presented in the study are included in the article/supplementary material, further inquiries can be directed to the corresponding author.

## Supplementary Materials

The following supporting information can be downloaded at https://www.mdpi.com/article/10.3390/children11040471/s1. Table S1: The questionnaire used in the current study.
