# Supplementary material for: Intertemporal Improvement in Physicians’ Perceptions of the Short-Term Adverse Outcomes of Neonatal Pain: Results of a Two-Time-Point National Survey
Source: Children (Basel). 2024 Apr 15;11(4):471. doi: 10.3390/children11040471 (PMC11049171; doi:10.3390/children11040471)
Supplement: Supplementary file 1 [file children-11-00471-s001.zip › children-2951236-SI.pdf]

SUPPLEMENTARY MATERIAL

Table S1. The questionnaire used in the current study

1ST DEPARTMENT OF NEONATOLOGY AND INTENSIVE NEONATAL CARE

QUESTIONNAIRE CONCERNING PHYSICIANS' PRECEPTIONS ON POTENTIAL SHORT-TERM ADVERSE EFFECTS OF ANALGESIA AND SEDATION IN NEONATAL INTENSIVE CARE UNITS.

PERSONAL DATA (not identifying responder's ID)

Sex: male ..☐……… female …☐…….

Type of NICU where you work: A. Public Hosp ..☐ B. Private Hosp.☐

Work experience: A. qualified neonatologist ..☐. B. pediatrician working in NICU for at least 3 years .☐.. C. Fellows .☐.

Years working in the NICU: ………………….

Questionnaire regarding your views on potential short term adverse effect of pain on the neonate

1A. Do you believe that neonates can feel pain?

YES ☐ NO ☐ □

1.B. Do you believe that pain may have adverse effects on the neonate?

YES ☐ NO ☐ □

1.C. If your reply is yes to the 1.B. question, which of following organ/system – specific adverse effect might be?? Check (YES) for positive answers.

	Domains and items	YES	NO
**1**	**CNS**		
1.1.	change of cerebral blood flow		
1.2	Cerebral hemorrhage		
1.3	Increased intracranial pressure		

1.4	Increased stress responses		
**2**	**Cardiovascular system**		
2.1	Hypertension		
2.2	Tachycardia		
2.3	Bradycardia		
2,4	Heart rate fluctuation		
2.5	Pulmonary hypertension		
2.6	Circulatory collapse		
**3**	**Respiratory system**		
3.1	Irregular breathing pattern		
3.2	Apneic spells		
3.3	Respiratory distress		
3.4	Oxygen desaturations		
3.5	Hypoxemia		
3.6	Ventilator asynchrony		
3.7	Increase duration of mechanical ventilation		
3.8	Pneumothorax		
**4**	**Other systems (growth, metabolism, endocrine s.)**		
4.1	Deterioration of the clinical condition		
4.2	Hormonal & metabolic derangement		
4.3	Slow growth rate		
